# CdS Nanocubes Adorned by Graphitic C_3_N_4_ Nanoparticles for Hydrogenating Nitroaromatics: A Route of Visible-Light-Induced Heterogeneous Hollow Structural Photocatalysis

**DOI:** 10.3390/molecules27175438

**Published:** 2022-08-25

**Authors:** Zhi-Yu Liang, Feng Chen, Ren-Kun Huang, Wang-Jun Huang, Ying Wang, Ruo-Wen Liang, Gui-Yang Yan

**Affiliations:** 1 Province University Key Laboratory of Green Energy and Environment Catalysis, Ningde Normal University, Ningde 352100, China; 2 Fujian Provincial Key Laboratory of Featured Materials in Biochemical Industry, Ningde Normal University, Ningde 352100, China; 3 College of Environmental Science and Engineering, Fujian Normal University, Fuzhou 350117, China

**Keywords:** heterogeneous hollow structural materials, CdS nanocubes, graphitic carbon nitride, photoreduction catalysis

## Abstract

Modulating the transport route of photogenerated carriers on hollow cadmium sulfide without changing its intrinsic structure remains fascinating and challenging. In this work, a series of well-defined heterogeneous hollow structural materials consisting of CdS hollow nanocubes (CdS NCs) and graphitic C_3_N_4_ nanoparticles (CN NPs) were strategically designed and fabricated according to an electrostatic interaction approach. It was found that such CN NPs/CdS NCs still retained the hollow structure after CN NP adorning and demonstrated versatile and remarkably boosted photoreduction performance. Specifically, under visible light irradiation (λ ≥ 420 nm), the hydrogenation ratio over 2CN NPs/CdS NCs (the mass ratio of CN NPs to CdS NCs is controlled to be 2%) toward nitrobenzene, *p*-nitroaniline, *p*-nitrotoluene, *p*-nitrophenol, and *p*-nitrochlorobenzene can be increased to 100%, 99.9%, 83.2%, 93.6%, and 98.2%, respectively. In addition, based on the results of photoelectrochemical performances, the 2CN NPs/CdS NCs reach a 0.46% applied bias photo-to-current efficiency, indicating that the combination with CN NPs can indeed improve the migration and motion behavior of photogenerated carriers, besides ameliorating the photocorrosion and prolonging the lifetime of CdS NCs.

## 1. Introduction

Recently, micro- and nanomaterials with dimensions in the nanometer and submicron scales have attracted significant interest [1]. For example, in terms of the microstructure of materials, researchers have developed a myriad of micro- and nanomaterials with different morphologies, including nanoparticles [2], nanorods [3], nanosheets [4], and hollow structures [5]. Among the many morphologies, hollow structural micro- and nanomaterials possess a larger specific surface area, lower density, shorter charge transport distance, and higher loading capacity and have been successfully used in many fields, such as photocatalysis [6,7,8]. In particular, hollow CdS-based photocatalysts, which possess outstanding photocatalytic activity toward environmental remediation and energy production under visible light irradiation, have surpassed their solid counterparts in many aspects, such as larger surface area and more exposed active sites to trigger many redox reactions [9,10]. All of these are coupled with the high capacity for light absorption through light reflection and scattering in the inner cavity, as the hollow structure endows the CdS with enhanced photocatalytic properties. Therefore, hollow CdS-based photocatalysts have broad application prospects in the field of photocatalysis and are worthy of our further study.

Regardless of the structures of pure hollow CdS-based photocatalysts, the practical application remains restricted by the severe photocorrosion and high recombination rate of photogenerated charges [11]; thus, it is necessary to find a suitable co-catalyst to improve the shortcoming of severe photocorrosion. In the in-depth study of the morphology, size, and composition of hollow structural materials, heterogeneous hollow structural materials, which are formed by the combination of different compositions or different crystalline phases as units, were extremely fascinating due to the abundance of design approaches and the superiority of performance control [12,13,14,15]. Compared with conventional single-component hollow structural materials, heterogeneous hollow structural materials not only have the traditional advantages of hollow structures, but also show rich functionalities in energy conversion and storage, catalysis, and drug delivery due to the synergistic effect between different components [12,16,17,18].

Theoretically, the hollow structure can rapidly transport the generated electrons and holes to two counterparts by enhancing the built-in electric field at the shell level, avoiding the recombination of electrons and holes, and improving the photocatalytic activity [19,20,21,22]. Hitherto, multiple efforts have been devoted to developing heterogeneous hollow structural material-based photocatalysts. In particular, the effective transfer of photogenerated holes from the valence band of CdS would be an ideal strategy to deal with the photocorrosion problem and improve the stability of CdS. Fortunately, graphitic carbon nitride (g-C_3_N_4_) has a suitable energy level for constructing a heterostructure with CdS to afford an effective separation and transfer of photogenerated charges [23,24,25,26,27,28]. Thanks to its rigid heptazine ring structure, π-conjugated electronic structure, and high condensation degree, g-C_3_N_4_ possesses many advantages in building the heterostructure, including excellent physicochemical stability and attractive electronic structure combined with an appropriate band gap (~2.7 eV). In addition, g-C_3_N_4_ is readily available and can be easily prepared from cheap crude materials such as urea [29,30,31]. However, due to the irregular size and low photocatalytic activity caused by the buckling nature of bulk g-C_3_N_4_, it is necessary to design a favorable and facile morphology of g-C_3_N_4_ for constructing a hollow CdS-based heterogeneous photocatalyst.

In this work, we synthesized a series of heterogeneous hollow structural materials constructed by hollow CdS nanocubes (CdS NCs) and g-C_3_N_4_ nanoparticles (CN NPs), in which CN NPs with abundant active sites were employed to reduce the photocorrosion and enhance the photocatalytic performance of CdS NCs. Consequently, the resultant CN NP/CdS NC heterogeneous hollow structural materials demonstrated superior photoactivities to the single counterparts toward hydrogenation of nitroaromatics to amino derivatives under visible light irradiation. In addition, according to the variety of photoelectrochemical performances, the CN NPs worked as a competent and suitable co-catalyst to separate the photogenerated charges and improve the stability of CdS NCs; furthermore, they prolong the lifetime of the photogenerated charges. The possible mechanism for the enhancement of photocatalytic efficiency was studied in particular.

## 2. Results

### 2.1. Characterizations of CN NPs/CdS NCs

**Figure 1:**
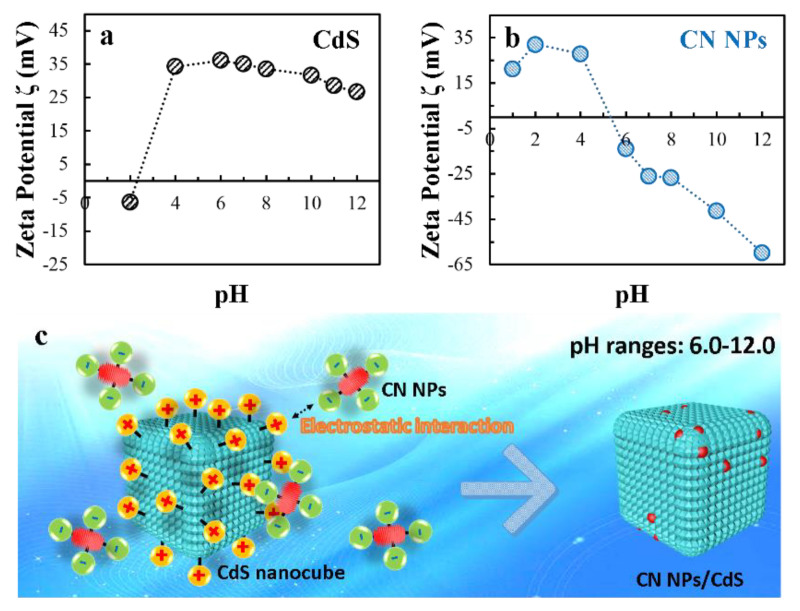
Zeta potential test of (**a**) CdS NCs and (**b**) CN NPs. (**c**) Schematic diagram of synthesis approach for CN NPs/CdS NCs by electrostatic interaction.

The detailed synthesis approach for CN NPs/CdS NCs is schematically demonstrated in Figure 1. Based on the results of the zeta potential test, the CdS NC aqueous solution is positively charged within the range of pH from 3 to 12 (Figure 1a). On the other hand, it is noteworthy that the CN NP solution (Figure 1b) shows a negatively charged status within the pH range from 6 to 12. In this regard, when the solution has a pH of more than 6, the CN NPs with a negative charge would be spontaneously and uniformly self-assembled on the surface of CdS NCs with a positive charge by electrostatic interaction.

**Figure 2:**
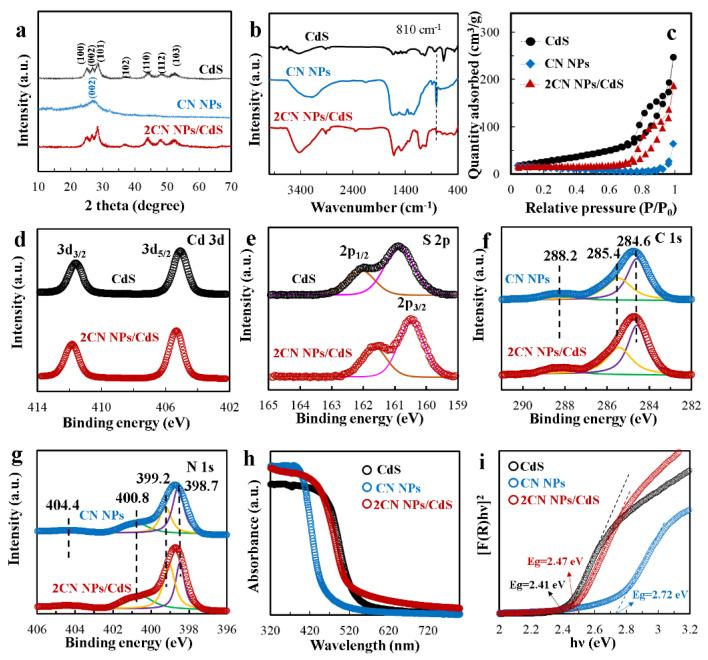
(**a**) XRD patterns, (**b**) FTIR spectra, and (**c**) BET measures of the CdS NCs, CN NPs, and 2CN/CdS NCs. Survey and high-resolution XPS spectra of (**d**) Cd 3d, (**e**) S 2p, (**f**) C 1s, and (**g**) N 1s for CdS NCs, CN NPs, and 2CN NPs/CdS NCs. (**h**) UV-Vis diffuse reflectance spectra of CdS NCs, CN NPs, and 2CN NPs/CdS NCs with (**i**) band gap determinations based on the Kubelka–Munk method.

X-ray diffraction (XRD) was performed to verify the crystalline phases. CdS NCs, CN NPs, and 2CN NPs/CdS NCs (the mass ratio of CN NPs to CdS NCs is controlled to be 2%) were selected for a concise comparison, as shown in Figure 2a. Hexagonal wurtzite CdS (JCPDS 41-1049) could be found in the pristine CdS NCs and the 2CN NPs/CdS NCs [9,10,32,33,34]. The peak at 2θ = 27.2° in the XRD of the CN NPs (JCPDS 87-1526) was ascribed to the crystallographic plane (002) of graphite [31,35,36,37]. Note that this characteristic peak of g-C_3_N_4_ disappeared when the CN NPs hybridized with the CdS NCs, which may be ascribed to the rather low loading amount of CN NPs compared with CdS NCs in the composite; the peak of g-C_3_N_4_ was probably covered by the CdS peaks.

The Fourier transform infrared spectroscopy (FTIR) spectra of different samples were inspected to verify the integration of CN NPs and CdS NCs in the composites. Figure 2b shows that the absorption bands at 625, 1078, and 1338 cm^−1^, attributable to the vibration mode of Cd–S [38,39], could be observed both in the spectrum of CdS NCs and CN NPs/CdS NCs. Meanwhile, the absorption in the region of 1240–1680 cm^−1^, corresponding to the stretching modes of the heterocycles in CN NPs, was also observed both in the CN NPs and the 2CN NP/CdS NC composite [40,41]. Therefore, in combination, the XRD and FTIR tests indicated that the integration of the CN NPs and CdS NCs was successful according to the electrostatic interaction.

Additionally, Brunauer–Emmett–Teller (BET) measurements were performed to evaluate the specific surface areas of CdS NCs, CN NPs, and 2CN NPs/CdS NCs. As illustrated in Figure 2c, specific surface areas of CdS NCs, CN NPs, and 2CN NPs/CdS NCs were 97.5, 6.3, and 45.3 m^2^/g, respectively. It is noteworthy that after combining with CN NPs, the specific surface area of 2CN NPs/CdS NCs is dramatically decreased, which can be attributed to the integration of CN NPs on the CdS NC substrates shielding the pores of CdS NC frameworks.

Elemental chemical states of CN NPs, CdS NCs, and 2CN NPs/CdS NCs were determined by X-ray photoelectron spectroscopy (XPS). As shown in Figure 2d–g, the high-resolution spectrum of Cd 3d suggests the presence of Cd^2+^. Additionally, the characterized peaks located at 161.3 eV for S 2p_3/2_ and 162.7 eV for S (Figure 2e) can be ascribed to S^2−^ [42,43]. Peaks centered at 284.6, 285.4, and 288.2 eV in the C 1s spectrum corresponded to C-C/C-H, C-OH, and N-C=N, respectively. Finally, the peaks at 398.7, 399.2, and 400.8 eV of the N 1s spectrum are attributable to the nitrogen species of C-N=C, N-(C)_3_, and C-N-H groups, respectively [44,45]. As such, XPS results signify again the successful combination of CN NPs and CdS NSs in the composites.

To determine the optical properties of as-prepared samples, diffuse reflectance spectroscopy (DRS) was performed. Figure 2h shows that the CdS NCs, CN NPs, and 2CN NPs/CdS NCs had substantial absorption in the visible region, which could be attributed to the intrinsic absorption properties of CdS and g-C_3_N_4_. Additionally, according to the Kubelka–Munk method [46,47], the band gap of CdS NCs, CN NPs, and 2CN NPs/CdS NCs could be roughly determined as 2.41, 2.72, and 2.47 eV by drawing a tangent line on the plots of (αhν)^2^ vs. hν, as shown in Figure 2i. The results of DRS further suggested that the energy band structures of as-prepared samples are suitable for absorption of visible light.

**Figure 3:**
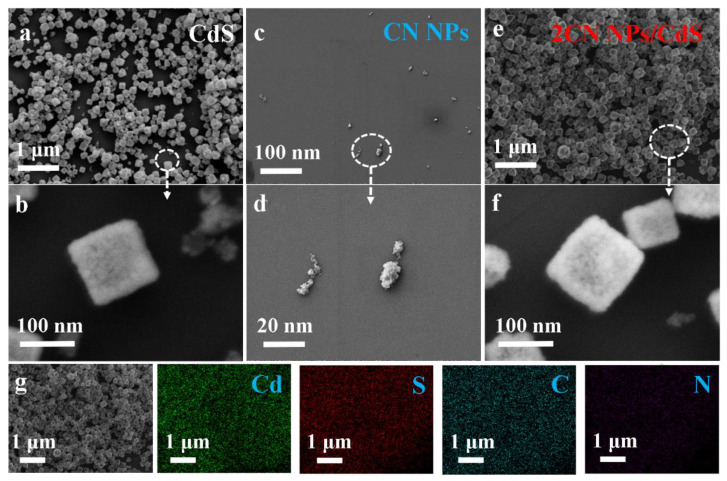
SEM images of the (**a**,**b**) CdS NCs, (**c**,**d**) CN NPs, and (**e**,**f**) 2CN NPs/CdS NCs. (**g**) EDS mapping analysis of Cd, S, C, and N elements over 2CN NPs/CdS NCs.

The morphologies of CdS NCs, CN NPs, and 2CN NPs/CdS NCs were investigated by FESEM. As demonstrated in Figure 3a,b, uniform and neat nanocubes were observed. Figure 3c,d show the SEM image of CN NPs, which ultrasmall nanoparticles with a mean diameter of ca. 5–20 nm. It is worth noting that the framework of CdS NCs had no change after combination with CN NPs, implying that electrostatic interaction can be considered as a mild and efficient assembly route, as shown in Figure 3e,f. Additionally, EDS mapping analysis was also performed (Figure 3g) to elucidate the elemental distribution in the nanocomposites, confirming the presence of the Cd, S, C, and N elements in the 2CN NPs/CdS NCs. Based on the SEM and EDS results, it is confirmed that the CN NPs were tightly integrated on the surface of CdS NCs after electrostatic self-assembly.

**Figure 4:**
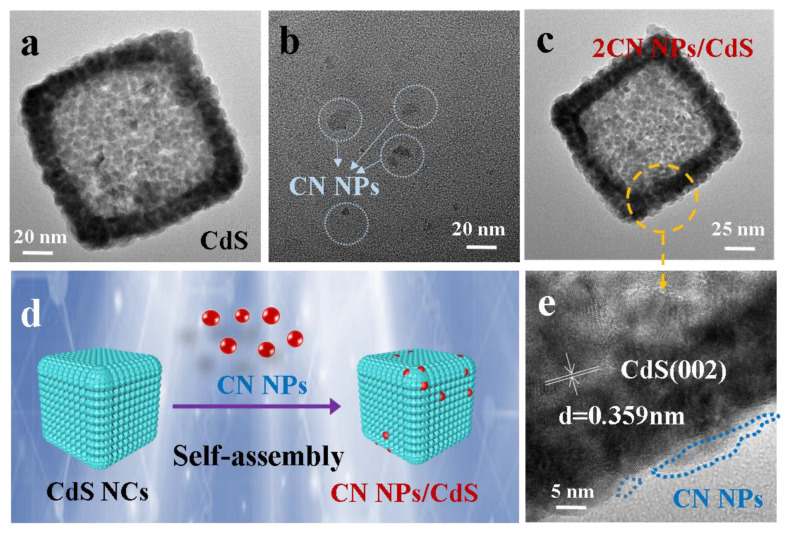
TEM images of (**a**) CdS NCs, (**b**) CN NPs, and (**c**) the 2CN NP/CdS NC heterogeneous hollow photocatalyst; (**d**) schematic illustration of the flowchart for fabricating the CN NPs/CdS NCs; (**e**) HRTEM image of the 2CN NP/CdS NC heterogeneous hollow photocatalyst.

Transmission electron microscopy (TEM) measurements were performed to inspect the morphologies of the CdS NCs, CN NPs, and 2CN NPs/CdS NCs (Figure 4a). In the TEM images of CdS NCs, a polycrystalline structure of hollow nanocubes with a mean diameter of 100 nm could be observed. It was previously demonstrated that the complex hollow nanostructure of CdS possessed superior photocatalytic activity to the other forms of CdS. The TEM images of the CN NPs had many ultrasmall nanoparticles with a diameter of ca. 5–20 nm (Figure 4b). Therefore, given the surface charge properties of these assembly units, it could be reasonably speculated that the negatively charged CN NPs would spontaneously and uniformly self-assemble on the positively charged hollow CdS NCs to afford intimate interfacial contact under electrostatic interaction (Figure 4d). In 2CN NPs/CdS NCs, lattice spacing (0.359 nm) was accurately assigned to the (002) crystallographic plane of hexagonal CdS [20,37,48]. The blue circles in Figure 4e mark some non-crystalline CN NPs that loaded on the CdS NCs surface uniformly and intimately, indicating the successful synthesis of heterogeneous hollow structural materials by combination of CdS NCs and CN NPs.

### 2.2. Photocatalytic Performances of CN NPs/CdS NCs

**Figure 5:**
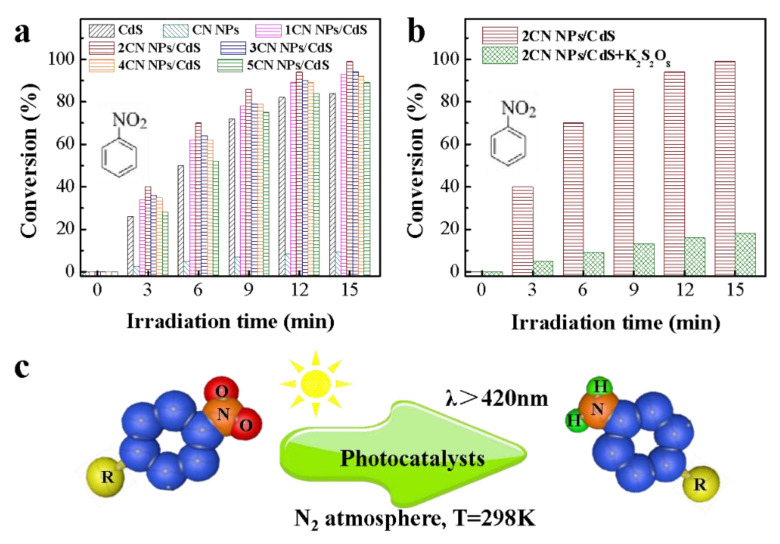
(**a**) Photocatalytic performance of CdS NCs, CN NPs, and xCN NP/CdS NC (x is the mass ratio of CN NPs to CdS NCs) heterogeneous hollow photocatalysts with different CN NP loading amounts under visible light irradiation with N_2_ aeration. (**b**) Photoactivities of 2CN NPs/CdS NCs when adding K_2_S_2_O_8_ as electron quench under visible light irradiation with N_2_ aeration. (**c**) Typical reaction model of converting aromatic nitro compounds to aromatic amines under the experimental environment.

The photocatalytic performance of the substrates was probed under visible light (λ > 420 nm) irradiation at ambient conditions by the selective anaerobic photocatalytic hydrogenation of aromatic nitro compounds to the corresponding amino compounds. An example is the photocatalytic hydrogenation of nitrobenzene to aniline. According to UV-Vis spectroscopy, the absorption peak at 273 nm was attributed to nitrobenzene, and the peaks at 238 nm were ascribed to aniline. Figure 5a shows that the CN NPs had a low photoactivity and reduced only ca. 9.4% of the nitrobenzene after irradiation for 15 min, whereas the CdS NCs reduced ca. 84.1% of the nitrobenzene in the same irradiation time. When the CN NPs and CdS NCs were combined, the CN NPs/CdS NC heterogeneous hollow photocatalysts exhibited strongly enhanced photocatalytic performances. Among the different CN NPs loading amounts, the photoactivity of 2CN NP/CdS heterogeneous hollow photocatalyst demonstrated the optimal photoactivity (~100%) in comparison with the other counterparts. Consequently, the results indicated that the integration of CdS NCs with CN NPs in a rational way contributes to enhancing performance. Furthermore, to demonstrate the critical role of photoelectrons in initiating the progress of reducing nitrobenzene, K_2_S_2_O_8_ was used as an electron quench. Figure 5b shows that the photoactivity decreased remarkably for 2CN NPs/CdS under the same reaction conditions, therefore indicating the crucial role of in situ photoexcited electrons in initiating the reaction.

**Figure 6:**
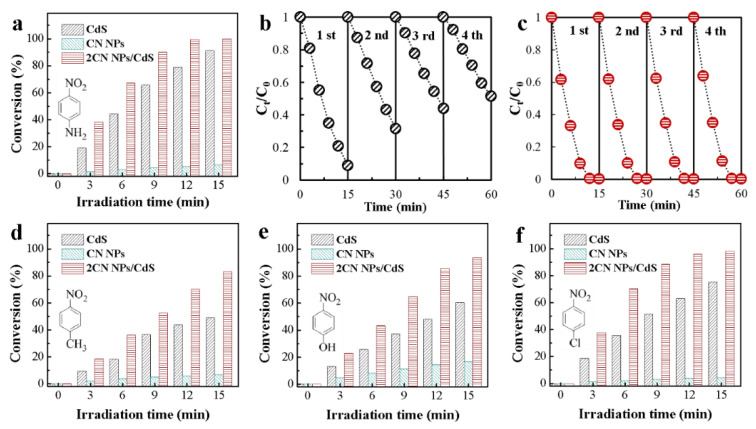
Photocatalytic performance toward reducing (**a**) *p*-nitroaniline, (**d**) *p*-nitrotoluene, (**e**) *p*-nitrophenol, and (**f**) *p*-nitrochlorobenzene over CdS NCs, CN NPs, and 2CN NPs/CdS under visible light irradiation with N_2_ aeration. Cyclic experiments for photocatalytic reducing of *p*-nitroaniline over (**b**) CdS NCs and (**c**) 2CN NP/CdS NC heterogeneous hollow photocatalyst under the experimental environment.

Apart from nitrobenzene, the selection of other aromatic nitro compounds such as *p*-nitroaniline, *p*-nitrotoluene, *p*-nitrophenol, and *p*-nitrochlorobenzene over CdS NCs, CN NPs, and 2CN NP/CdS NC heterogeneous hollow photocatalyst were also explored. As shown in Figure 6a–f, compared with CdS NCs and CN NPs, 2CN NPs/CdS still shows the optimal photocatalytic activity for hydrogenation of aromatic nitro compounds. The photocatalytic performance toward *p*-nitroaniline, *p*-nitrotoluene, *p*-nitrophenol, and *p*-nitrochlorobenzene over 2CN NPs/CdS was found to be 99.9%, 83.2%, 93.6%, and 98.2%, respectively. Hence, the integration of CdS NCs and CN NPs in an appropriate approach will be beneficial for enhancing the photocatalytic performances under visible light. Meanwhile, cycling experiments were carried out to demonstrate that CN NPs can improve the photostability of CdS. As shown in Figure 6b,c, the 2CN NPs/CdS NCs maintained excellent photostability (97.7%) for the photocatalytic hydrogenation of *p*-nitroaniline after four consecutive cycling experiments. However, the photocatalytic hydrogenation activity of CdS NCs was reduced to only 48.6%. It was clearly shown that CN NPs will ameliorate the photocorrosion of the CdS NCs after integration.

Table 1 summarizes a comparative study of different photocatalysts reported in recent years toward the hydrogenation of nitrobenzene. It is clearly shown that the as-prepared samples showed excellent performance toward the hydrogenation of nitrobenzene.

### 2.3. Photoelectrochemical (PEC) Measurements

**Figure 7:**
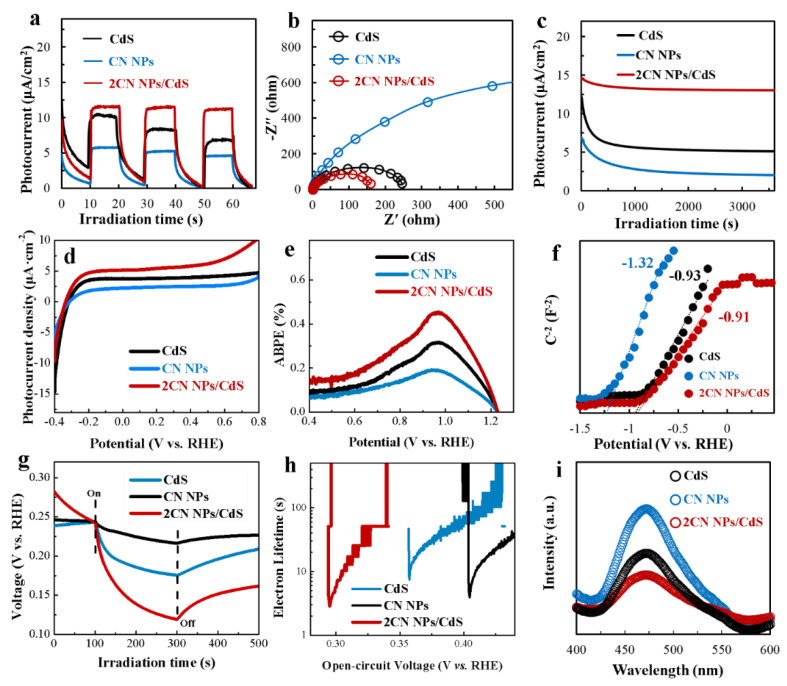
(**a**) Transient photocurrent responses with a 0.1 V vs. RHE bias voltage, (**b**) EIS results, (**c**) photostability, (**d**) LSV (scan rate: 5 mV/s), (**e**) ABPE curves based on the LSV results, (**f**) Mott–Schottky plots, and (**g**) OCVD curves of CdS NCs, CN NPs, and 2CN NP/CdS NC heterogeneous hollow photocatalyst under visible light irradiation. (**h**) Electron lifetime based on the results of OCVD curves and (**i**) PL spectra of the as-prepared materials with an excitation wavelength of 325 nm.

Photoelectrochemical (PEC) measurements were performed to observe the PEC properties of CdS NCs, CN NPs, and CN NPs/CdS NC heterogeneous hollow photocatalysts. Figure 7a shows the transient photocurrent responses of CdS NCs, CN NPs, and the 2CN NP/CdS NC heterogeneous hollow photocatalyst under visible light, following the subsequence of 2CN NPs/CdS NCs > CdS NCs > CN NPs, which is in line with the photocatalytic performances. Additionally, electrochemical impedance spectroscopy (EIS) was performed to evaluate the separation efficiency of photogenerated charge carriers in the interfaces. Generally, a smaller Nyquist plot semicircle radius represents a faster interfacial charge transfer rate [54]. As shown in Figure 7b, the EIS Nyquist plot of the 2CN NP/CdS NC heterogeneous hollow photocatalysts exhibits a smaller semicircular arc radius under visible light than that of CdS NCs and CN NPs, implying that the interface of the 2CN NP/CdS NC heterogeneous hollow photocatalyst will facilitate the moving and transmission of photogenerated electrons. Furthermore, after 1 h of continuous irradiation, the 2CN CdS NCs exhibit more photostability than CdS NCs (Figure 7c), indicating that the CN NPs can indeed ameliorate the photocorrosion of CdS NCs.

Additionally, linear sweep photovoltammetry (LSV) can be used to assess the kinetics of charge motion. As shown in Figure 7d, the photocurrent of the 2CN CdS NC heterogeneous hollow photocatalyst increases in the high bias voltage region, indicating that 2CN CdS NCs have a beneficial interface for charge movement [55]. For the results of LSV, the conversion efficiency can be estimated by calculating the applied bias photo-to-current efficiency (ABPE, η) with Equation (1).

(1) $$\eta =\frac{I\left(1.23-\left|V\right|\right)}{P}$$

where *I* is the photocurrent measured by LSV, *V* is the bias, and *P* is the intensity of irradiation light (about 20 mW·cm^−2^). The results are reflected in Figure 7e, and the 2CN CdS NCs have the highest efficiency, about 0.46%. Notably, the results of LSV and ABPE are quite in line with the photoactivity performances, consistently confirming that the motion behavior of photogenerated charges will be significantly promoted after adorning the surface of CdS nanocubes with CN NPs.

Furthermore, the CB of semiconductors could be determined by Mott–Schottky (M-S) measurements, as shown in Figure 7f, flat-band potentials of CdS NCs and CN NPs are determined to be −0.93 and −1.22 eV (vs. RHE). Thus, combined with the results of UV-Vis DRS, the VB location of CdS NSs and CN NPs can be roughly calculated as +1.48 V and +1.40 V, respectively. Such band structures are beneficial for the transmission of the photogenerated electrons from the CB of CN NPs to the CB of CdS NCs, as well as the photogenerated holes from the VB of CdS NCs to the VB of CN NPs, thus ameliorating the photocorrosion of CdS NCs.

Additionally, the recombination kinetics and lifetime of charge could be estimated by monitoring the open-circuit photovoltage decay (OCVD) tests [49]. The 2CN CdS NC heterogeneous hollow photocatalyst exhibits a larger photovoltage than CdS NCs and CN NPs, implying the lowest recombination of charge, as shown in Figure 7g. Based on the OCVD results, the logarithmic graph of electron lifetime vs. open-circuit voltage could be calculated by Equation (2),

(2) $$\tau =\frac{-k_{B}T}{e}{\left(\frac{dV_{oc}}{dt}\right)}^{-1}$$

where electron lifetime, thermal energy, electron charge (~1.602 × 10^−19^ C), time-dependent photovoltage, and time are abbreviated as τ, *k_B_T*, *e*, *V_oc_*, and *t*. The calculated results are reflected in Figure 7h. The 2CN NP/CdS NC heterogeneous hollow photocatalyst shows a longer electron lifetime. Obviously, the result is in line with the other PEC tests. Such a conclusion can be further confirmed by the photoluminescence (PL) intensity test; as shown in Figure 7i, the lowest intensity of the 2CN NP/CdS NC heterogeneous hollow photocatalyst indicates an optimal charge separation efficiency.

**Scheme 1:**
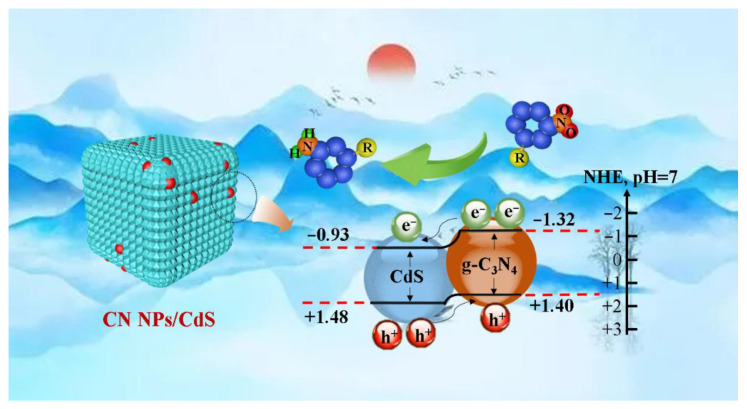
Schematic illustration of the photocatalytic hydrogenation mechanism of the CN NP/CdS NC heterogeneous hollow photocatalysts.

Based on the above analysis, the photocatalytic mechanism of the CN/CdS NC heterogeneous hollow photocatalyst was proposed, as shown in Scheme 1. Under the irradiation of visible light, both CN NPs and CdS NCs were excited to produce the electron–hole pairs. The electrons in the conduction band of the CN NPs easily and immediately migrated into the conduction band of the CdS NCs because (1) CN NPs had a more negative electronic potential in their conduction band compared with CdS NCs and (2) the interfacial integration between CN NPs and CdS NCs was highly intimate. Meanwhile, the holes in the valence band of the CdS NCs spontaneously flew into the valence band of the CN NPs, which effectively prevented the photocorrosion of the CdS NCs and prolonged their lifetime. As for the photoreduction, the electrons in the conduction band of the CdS NCs efficiently reduced the aromatic nitro compounds to the corresponding amines under the N_2_ atmosphere.

## 3. Materials and Methods

### 3.1. Materials

Urea, sulfuric acid (H_2_SO_4_), nitric acid (HNO_3_), sodium hydroxide (NaOH), hydrochloric acid (HCl), ammonium hydroxide (NH_3_·H_2_O), polyvinyl pyrrolidone K-30, silver nitrate (AgNO_3_), sodium citrate, sodium borohydride (NaBH_4_), ferric chloride (FeCl_3_), sodium sulfide (Na_2_S), cadmium nitrate (Cd(NO_3_)_2_), tributylphosphine, concentrated NH_3_ H_2_O, potassium hexacyanoferrate (III) (K_3_[Fe(CN)_6_]), potassium hexacyanoferrate (II) (K_4_[Fe(CN)_6_]), sodium sulfate (Na_2_SO_4_), ethanol (C_2_H_5_OH), deionized water (DI H_2_O, Millipore, 18.2 MΩ cm resistivity), ammonium formate (HCO_2_NH_4_), potassium persulfate (KPS, K_2_S_2_O_8_), nitrobenzene, *p*-nitroaniline, *p*-nitrophenol, *p*-nitrotoluene, and *p*-nitrochlorobenzene were are analytical grade and used as received without further purification.

### 3.2. Synthesis

#### 3.2.1. Synthesis of CdS Nanocubes (CdS NCs)

The CdS nanocubes were prepared as follows [10,32,33]: polyvinyl pyrrolidone K-30 (84 mg) and aqueous AgNO_3_ (50 mmol/L, 1 mL) were added into a solution of aqueous sodium citrate (10 mmol/L, 40 mL) before the sequential addition of aqueous NaBH_4_ (10 mmol/L, 1 mL), concentrated HCl (200 μL), and aqueous FeCl_3_ (0.1 mol/L, 50 μL) under constant strong stirring. The mixed solution was maintained at 30 °C for 16 h to form a homogeneous suspension, to which aqueous Na_2_S (0.1 mol/L, 1 mL) was added. The precipitates were collected, washed three times with methanol, and dispersed in methanol (4 mL). The solution of Cd(NO_3_)_2_ in methanol (50 mmol/L, 1 mL) and polyvinyl pyrrolidone K-30 (50 mg) were then added sequentially, and the mixture was stirred at 50 °C for 5 min. Finally, tributylphosphine (100 μL) was added and the mixture was kept at 50 °C for another 2 h. The precipitates were collected and washed three times with ethanol and deionized H_2_O to give CdS nanocubes (CdS NCs).

#### 3.2.2. Synthesis of g-C_3_N_4_ Nanoparticles (CN NPs)

Typically, urea (10 g) was heated from room temperature to 550 °C at 7 °C/min in a covered crucible for 2 h and then cooled to ambient temperature to obtain a yellow powder. The yellow powder (1 g) was added into a mixture solution including 20 mL H_2_SO_4_ and 20 mL HNO_3_ and then stirred at room temperature for 2 h. The mixture was then diluted with 1 L of deionized water and washed several times to give a white residue. The white residue was finally dispersed in 30 mL concentrated NH_3_·H_2_O and heated at 120 °C for 12 h in a 50 mL Teflon-lined autoclave, before being cooled to room temperature. The aqueous suspension was centrifuged at ~500 rpm to remove the precipitate and dialyzed to remove large-sized nanoparticles and NH_3_ molecules to provide an aqueous solution of g-C_3_N_4_ nanoparticles (marked as CN NPs).

#### 3.2.3. Preparation of g-C_3_N_4_ Nanoparticles/CdS Nanocubes (CN NPs/CdS NCs)

The g-C_3_N_4_ nanoparticles/CdS nanocubes (CN NPs/CdS NCs) were prepared by electrostatic self-assembly. CdS NCs (0.1 g) were firstly dispersed in ethanol (98 mL), and the aqueous g-CN NP solution was added dropwise; the pH of the mixture solution was adjusted to ~8.0 with aqueous NaOH (0.01 mol/L), and the mass ratio of CN NPs to CdS NCs was controlled to be 1%, 2%, 3%, 4%, and 5%. Finally, the precipitates were collected and washed three times with ethanol and deionized H_2_O to give the g-C_3_N_4_ nanoparticles/CdS nanocubes (CN NPs/CdS NCs).

### 3.3. Characterizations

The zeta potential was monitored with a Zeta Potential Analyzer (Omin, BIC, America). An X-ray diffraction (XRD, X’Pert Pro MPD) instrument equipped with Cu Kα radiation under 36 kV and 30 mA was employed to analyze the crystal structures. A TJ270-30A spectrophotometer (Tianjin Tuopu Instrument, China) was used to monitor the Fourier transform infrared spectroscopy (FT-IR) in the range of 400–4000 cm^−1^. The surface areas were determined from the nitrogen adsorption–desorption isotherm at 77.3 K by Brunauer–Emmett–Teller (BET, 3flex, Micromeritics, America). Field emission scanning electron microscopy (SEM, SUPRA 55, Carl Zeiss) was utilized to observe the morphologies. Transmission electron microscopy (TEM, FEI, America) was performed with an accelerating voltage of 200 kV. X-ray photoelectron spectroscopy (XPS, Escalab 250, Thermo Scientific, America) was analyzed with corrected by C 1s spectra of 284.6 eV. The UV-Vis diffuse reflectance spectroscopy (DRS) was performed on a Lambda 950 (PerkinElmer, America) by employing BaSO_4_ as a reflectance substrate. The photofluorescence (PL) spectra were measured on a Varian Cary Eclipse spectrometer with an excitation wavelength of 325 nm. Finally, all the PEC measurements were monitored using a three-electrode configuration on an electrochemical workstation (CHI-660C, Chenhua, China). Working electrodes: added the as-prepared photocatalysts (5 mg) into 0.5 mL ethanol, dripped on a 1 × 1 cm^2^ FTO glass, and finally dried the glass at 50 °C for 30 min to remove ethanol. Counter electrode: Pt foil. Reference electrode: Ag/AgCl. Electrolyte: Na_2_SO_4_ solution (1.0 mol·L^−1^).

### 3.4. Photoactivity Evolution

The photoactivity of the as-prepared samples was evaluated by reducing a series of aromatic nitro compounds. Typically, the catalyst (5 mg) and ammonium formate (10 mg, for quenching photogenerated holes) were evenly dispersed into the solution including the target nitro compound (30 mg L^−1^, 50 mL) with continuously bubbling N_2_. After stirring for 0.5 h in dark to reach an adsorption–desorption equilibrium, the mixture system was irradiated by a 500 W Xe lamp equipped with an optical filter (λ > 420 nm). Based on the equation of Lambert–Beer (Equation (3)), the photoactivity of the photocatalysts could be evaluated as follows:

(3) $$\mathrm{Conversion}=\left(\frac{C_{0}-C_{t}}{C_{0}}\right)\times 100\%$$

where *C*_0_ and *C_t_* represent the initial concentration and the time-dependent concentration, respectively.

## 4. Conclusions

In summary, we designed a series of well-fined heterogeneous hollow structural materials (CN NPs/CdS NCs) via a self-assembly approach by combining hollow CdS nanocubes and g-C_3_N_4_ nanoparticles. The intrinsically negatively charged CN NPs were uniformly and intimately interspersed on the positively charged hollow nanocubes of CdS NCs based on electrostatic interaction. The intimate integration of CN NPs with CdS NCs could effectively ameliorate the photocorrosion and enhance the performance of CdS NCs in photocatalytic hydrogenation. Under the irradiation of visible light, the CN NP/CdS NC heterogeneous hollow photocatalysts demonstrated markedly enhanced photocatalytic hydrogenation performance in the anaerobic photocatalytic hydrogenation of aromatic nitro compounds at ambient conditions; in particular, when the mass ratio of CN NPs to CdS NCs is 2%, the hydrogenation ratio over the heterogeneous hollow photocatalyst toward nitrobenzene, *p*-nitroaniline, *p*-nitrotoluene, *p*-nitrophenol, and *p*-nitrochlorobenzene can be increased to 100%, 99.9%, 83.2%, 93.6%, and 98.2%, respectively. The photogenerated electrons responsible for the photocatalytic hydrogenation process were unambiguously determined by systematic control experiments, based on which the photocatalytic mechanism was elucidated. Additionally, the 2CN NPs/CdS NCs reached the highest applied bias photo-to-current efficiency (0.46%) according to the PEC investigation. It is anticipated that our work may further inspire the rational design of heterogeneous hollow structural materials for highly efficient conversions making use of solar energy.

## Figures and Tables

**Table 1:** Comparison of the irradiation time and efficiency among various photocatalysts.

Materials	Irradiation Time (min)	Efficiency (%)	Ref.
2CN NPs/CdS NCs	15	100	This work
Ti_3_C_2_/Pd	150	99	[49]
1.2% PtO@Cr_2_O_3_	60	98	[50]
2% Bi_2_O_3_/Bi_2_WO_6_	40	93.1	[51]
20% rGO/NiCo_2_O_4_	60	100	[52]
1.2%PtO@ZnCr_2_O_4_	60	100	[53]

## Data Availability

Not applicable.
